# Social reward and nonsocial reward processing across the adult lifespan: An interim multi-echo fMRI and diffusion dataset

**DOI:** 10.1016/j.dib.2024.110810

**Published:** 2024-08-08

**Authors:** David V. Smith, Cooper J. Sharp, Abraham Dachs, James Wyngaarden, Daniel Sazhin, Yi Yang, Melanie Kos, Tia Tropea, Ishika Kohli, John A. Clithero, Ingrid Olson, Tania Giovannetti, Dominic Fareri, Johanna M. Jarcho

**Affiliations:** a Temple University, Philadelphia, PA 19122, USA; b University of Oregon, Eugene, OR 97401, USA; c Adelphi University, Garden City, NY 11530, USA

**Keywords:** Functional magnetic resonance imaging, Decision making, Brain, Reward processing, Striatum, Alzheimer's disease, Cognitive decline, Neurodegeneration, Connectivity

## Abstract

Social relationships change across the lifespan as social networks narrow and motivational priorities shift. These changes may affect, or reflect, differences in how older adults make decisions related to processing social and non-social rewards. While we have shown initial evidence that older adults have a blunted response to some features of social reward, further work in larger samples is needed to replicate our results and probe the extent to which age-related differences translate to real world consequences, such as financial exploitation. To address this gap, we are conducting a 5-year study funded by the National Institute on Aging (NIH R01-AG067011). Over the course of the funding period (2021–2026), this study seeks to: 1) characterize neural responses to social rewards across adulthood; 2) relate those responses to risk for financial exploitation and sociodemographic factors tied to risk; and 3) examine changes in risk for financial exploitation over time in healthy and vulnerable groups of older adults. This paper describes the preliminary release of data for the larger study. Adults (*N* = 114; 40 male / 70 female / 4 other or non-binary; 21–80 years of age *M* = 42.78, SD = 17.13) were recruited from the community to undergo multi-echo fMRI while completing tasks that measure brain function during social reward and decision making. Tasks probe neural response to social reward (e.g., peer vs. monetary feedback) and social context and closeness (e.g., sharing a monetary reward with a friend compared to a stranger). Neural response to social decision making is probed via economic trust and ultimatum games. Functional data are complimented by a T1 weighted anatomical scan and multi-shell diffusion-weighted imaging (DWI) to enable tractography and assess neurite orientation dispersion and density. Overall, this dataset has extensive potential for re-use, including leveraging multimodal neuroimaging data, within subject measures of fMRI data from different tasks – data features that are rarely seen in an adult lifespan dataset. Finally, the functional data will allow for developmentally sensitive cross-sectional analyses of differences in brain response to nuanced differences in reward contexts and outcomes (e.g., monetary vs. social; sharing winnings with a friend vs. stranger; stranger vs. computer).

Specifications Table

**Table:** 

Subject	Biological Sciences → Neuroscience: Cognitive
Specific subject area	Social and nonsocial reward systems
Type of data	anatomical brain images (compressed NIfTI files [.nii.gz]) diffusion brain images (compressed NIfTI files [.nii.gz]) functional brain images (compressed NIfTI files [.nii.gz]) experimental design information (tab-separated values [.tsv]) experiment meta-data (Java Script objection notation [.json])
Data collection	Anatomical, diffusion, and functional imaging data were collected from 114 participants (21–80 years old) using a 3.0 Tesla Siemens PRISMA MRI scanner and a 20-channel head coil at the Temple University Brain Imaging and Research Center. Anatomical data consisted of a T1-weighted scan with 1 × 1 × 1 mm voxels and a T2-weighted fluid attenuated inversion recovery scan with 0.86×0.86×3.00 mm voxels. Diffusion data were collected using a hybrid multi-shell diffusion weighted imaging sequence with 2 × 2 × 2 mm voxels. Multi-echo MRI were collected with four echoes (13.8, 31.54, 49.28, 67.02 ms) and simultaneous multislice (multiband = 3) with 2.7 × 2.7 × 2.7 mm voxels.
Data source location	Institution: Temple University Location: Philadelphia, Pennsylvania, USA
Data accessibility	Repository name: OpenNeuro Data identification number: https://doi.org/10.18112/openneuro.ds005123.v1.1.3 Direct URL to data: https://doi.org/10.18112/openneuro.ds005123.v1.1.3

## 1 Value of the Data

• These data are valuable because they include multiple tasks tapping into social reward processing and decision-making. Specifically, we use well-characterized tasks that probe neural responses to social reward (e.g., peer feedback) and social context and closeness (e.g., sharing a monetary reward with a friend compared to a stranger). In addition, we also use an economic trust game and the ultimatum game, which provide opportunities to examine social decision-making through the lens of computational modeling when combining choice data with non-choice data (e.g., response times) [1,2].
• Although data collection is ongoing, the interim dataset is based on a large (*N* = 114) community-based sample of racially diverse participants spanning a wide age range (21 to 80 years) and utilizes state-of-the-art multi-echo fMRI for increased signal to noise and reduced signal dropout in susceptibility regions. Notably, multi-echo data offers innovative opportunities for echo-time dependent denoising [3] and distortion correction [4].
• In addition to multi-echo fMRI, the dataset also includes multi-shell diffusion-weighted images (DWI), high-resolution T1-weighed (T1w) images, and T2-weighted fluid attenuated inversion recovery (T2w FLAIR) scans. These additional modalities increase the value of the dataset by creating opportunities for multi-modal analyses and linking structural and functional data. For example, researchers could use the FLAIR scans to quantify white matter hyperintensity burden [5] and assess how white matter hyperintensity burden is linked to neural responses to social reward processing and decision making.
• The fMRI data in this interim dataset were collected using two flip angles (20 and 50°). Although the lower flip angle was originally chosen based on prior work indicating that lower flip angles can reduce physiological noise [6], such effects have not been characterized in multi-echo data. Thus, this dataset provides an opportunity to characterize the effects of flip angle on BOLD signal under multiple task states and across various levels of physiological noise.
• This dataset includes tasks that are similar to other datasets that we have released on OpenNeuro [7,8], which creates opportunities for pooling data across studies and conducting well-powered analyses of social and nonsocial reward processing and decision making.

## 2 Background

Social relationships change across the lifespan as social networks narrow and motivational priorities shift to the present [9]. These changes can have significant impacts on how older adults make decisions and process rewards [10]. For example, recent work from our group has shown that the striatal response to reciprocated trust from close friends is blunted in older adults [11]. Yet, it remains unclear whether age-related differences in responses to social reward are linked to cognitive decline and risk for financial exploitation.

To address this gap, we are conducting a 5-year study funded by the National Institute on Aging (NIH R01-AG067011). Over the course of the funding period (2021–2026), this study seeks to: 1) characterize neural responses to social rewards across adulthood; 2) relate those responses to risk for financial exploitation and sociodemographic factors tied to risk; and 3) examine changes in risk for financial exploitation over time in healthy and vulnerable groups of older adults. The data described in this paper represents a preliminary release, mirroring the process of other large neuroimaging studies spanning multiple years [12,13]. Once the study concludes, we anticipate updating this data article with a greatly expanded sample size and expanded phenotypic data.

## 3 Data Description

These data are available as OpenNeuro Dataset ds005123 [14]. The dataset is composed of neuroimaging data from 114 participants. Each participant completed multiple tasks involving social and nonsocial reward processing while having blood-oxygenation-level-dependent (BOLD) responses recorded with multi-echo fMRI. The data are organized in accordance with the Brain Imaging Data Structure (BIDS) specification [15] using HeuDiConv [16]. Anatomical images (T1w and FLAIR) were defaced using PyDeface. As part of the BIDS standard format, all files are accompanied by .json sidecars that contain metadata regarding the file.

**Table:** 

Data Files	Description
participants.tsv	Basic demographics for each participant (gender, age at time of appointment, race, and ethnicity) as well as body mass index (BMI), self-reported handedness.
sub-*/sub-*_scans.tsv	Four column file that describes basic information regarding the images, including the order of acquisition
sub-*/anat/sub-*_T1w.nii.gz	NIfTI format T1 weighted anatomical
sub-*/anat/sub-*_FLAIR.nii.gz	NIfTI format FLAIR scan
sub-*/dwi/sub-*_dwi.bval	Diffusion-weighted imaging b-values
sub-*/dwi/sub-*_dwi.bvec	Diffusion-weighted imaging b-vectors
sub-*/dwi/sub-*_dwi.nii.gz	Diffusion-weighted imaging data
sub-*/fmap/sub-*_acq-bold_magnitude*.nii.gz	Magnitude component of B0 fieldmap
sub-*/fmap/sub-*_acq-bold_phasediff.nii.gz	Phase component of B0 fieldmap
sub-*/fmap/sub-*_acq-dwi_dir-AP_epi.nii.gz	Spin-echo fieldmap (anterior-posterior phase encoding)
sub-*/fmap/sub-*_acq-dwi_dir-PA_epi.nii.gz	Spin-echo fieldmap (posterior-anterior phase encoding)
sub-*/func/sub-*_task-*_run-*_part-mag_bold.nii.gz	BOLD data (magnitude data)
sub-*/func/sub-*_task-*_run-*_part-phase_bold.nii.gz	BOLD data (phase data)
sub-*/func/sub-*_task-*_run-*_sbref.nii.gz	Single-band reference image
sub-*/func/sub-*_task-*_run-*_events.tsv	Five column file describing the stimulus onset, duration, trial type, response time, and stim file. Note that one task (sharedreward) has a four column events file, only excluding stim file.

## 4 Experimental Design, Materials and Methods

### 4.1 Participants

We recruited 114 participants (40 male / 70 female / 4 other or non-binary) ranging from the ages of 21 to 80 years old (*M* = 42.78, SD = 17.13). Across the 114 participants, 68 were white, 24 were Black/African American, 11 were Asian, 8 were two or more races, and 3 preferred not to respond (see Table 1). Participants completed four counterbalanced neuroimaging tasks. All participants were recruited from advertisements targeting the greater Philadelphia metro area. Participants were paid $25 per hour for fMRI sessions and $15 an hour for all other data collection. Participants were also informed that they would be given an additional bonus based on task performance. All participants provided consent in accordance with the Institutional Review Board at Temple University.

**Table 1:** Demographic information.

	Male	Female	Non-Binary/Other	Total
Asian	0	10	1	**11**
Black / African American	13	10	1	**24**
White	22	45	1	**68**
Two or More Races / Race Not Described	3	4	1	**8**
Prefer Not to Respond	2	1	0	**3**
**Total**	**40**	**70**	**4**	**114**

### 4.2 Experimental tasks

Prior to coming to the neuroimaging session, participants completed a mock scan session to ensure compatibility with the neuroimaging environment (i.e., no claustrophobia, ability to keep head still).

During the neuroimaging session, each participant completed four tasks involving social decision making, social reward, and the receipt of monetary reward in different social contexts. These tasks involved different partners who were either known to the participant (i.e., a friend they selected) or unknown to the participant (i.e., an anonymous stranger). The order of tasks was counterbalanced across participants (scan/task order for each participant is described in each sub-*_scans.tsv file).

#### 4.2.1 Shared reward task

**Fig. 1:**
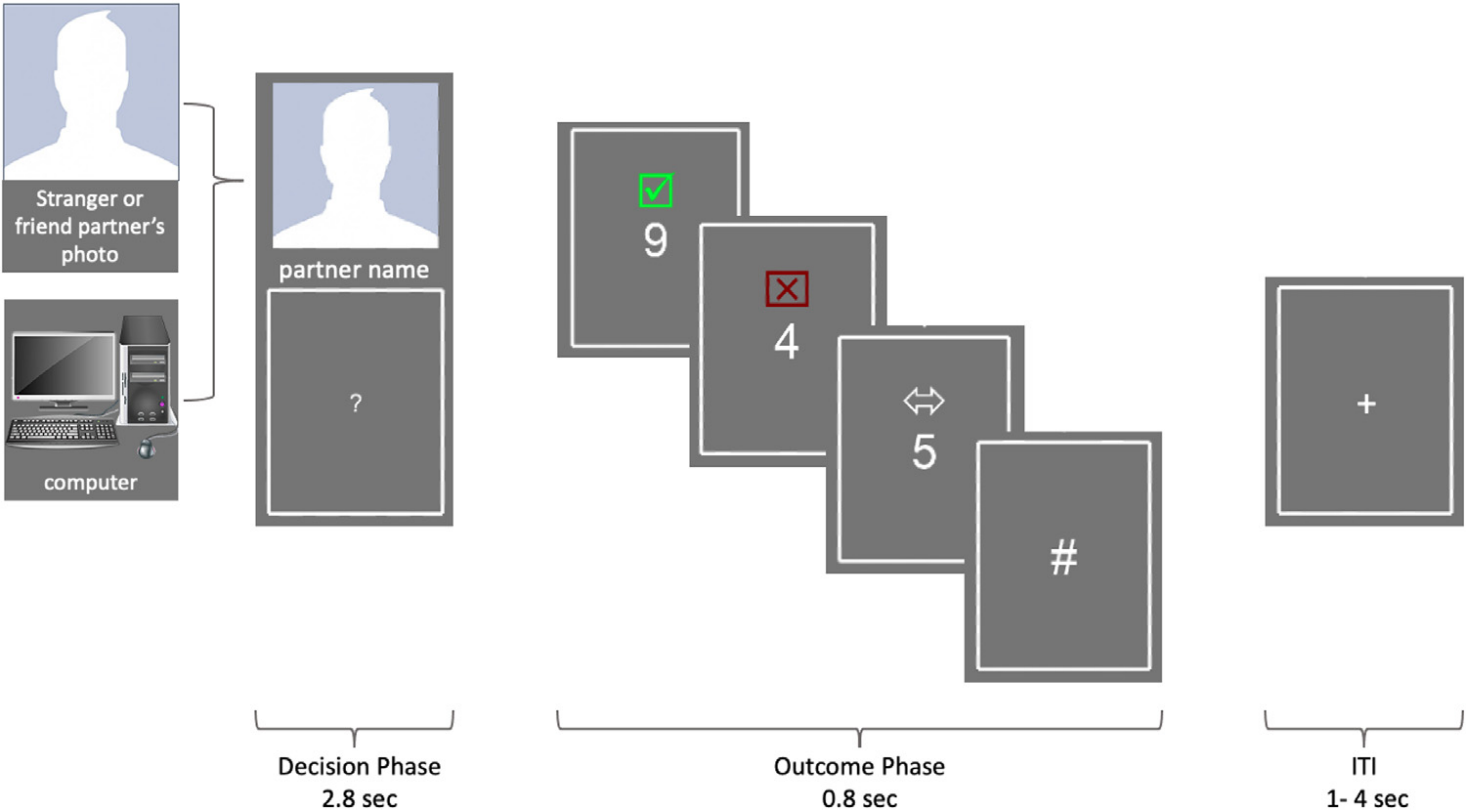
Shared Reward Task. During each trial, participants see the picture of their current partner and make guesses of the number on the card whether the number is above or below 5. Participants receive feedback in the form of a green check mark, a red cross mark, a white double-headed arrow, or a pound sign, signifying their guess is right, wrong, the number on the card is exactly 5, or they failed to make a guess in a timely manner, respectively. After the outcome, there is an intertrial interval that jitters between 1 – 4 s. After the interval, the participants will go to the next trial. Stranger partners’ photos were generated by AI.

Participants completed a card guessing task in three different social contexts (Fig. 1). Participants were instructed that all cards for the task had a value below ranging between 1 and 9, and their task on each trial was to indicate whether they thought the presented card would have a value above (i.e., 6, 7, 8, 9) or below 5 (i.e., 1, 2, 3, 4). Participants were presented with feedback on if they had made the correct guess (win) or an incorrect guess (loss). Some trials contained neutral feedback (i.e., the value of the card equaled 5). Participants played each round/trial with one of three potential partners: a friend (whose photo was submitted by the participant prior to the session), a stranger (a gender- and age-matched peer), or a computer. For every “win” decision, both the participant and their partner won $10 each, whereas for every “loss” decision, both the participant and their partner lost $5 each. No money was won or lost on trials where neutral outcomes were experienced.

#### 4.2.2 Social doors task

**Fig. 2:**
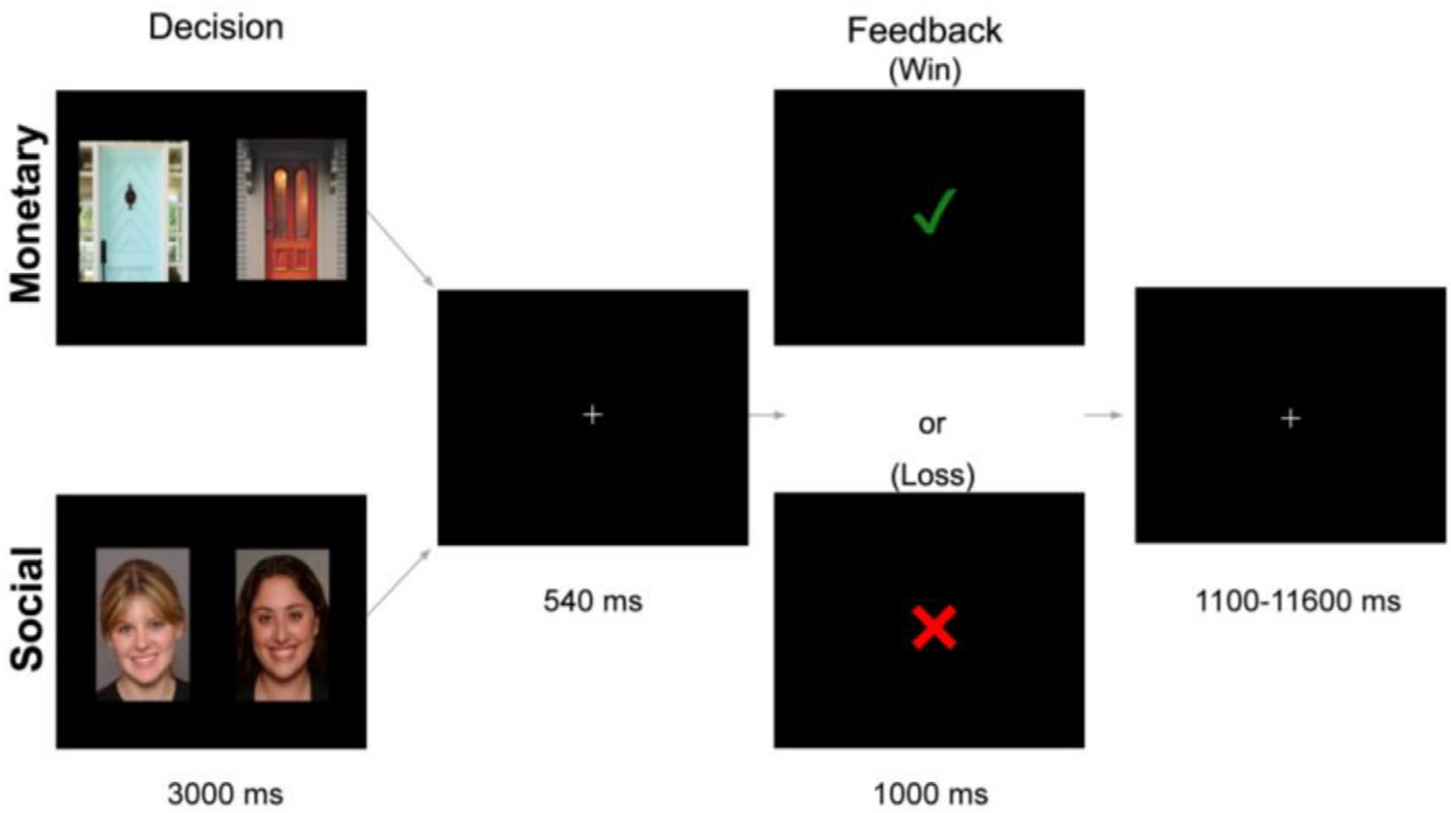
Social Doors Task. Participants make choices between two options on each trial: either between two doors (monetary condition) or the faces of two peers (social condition) in pursuit of a reward. Following a short delay, participants receive feedback in the form of a green checkmark signifying a win (monetary condition=$10 gain; social condition=positive peer feedback) or a red X indicating a loss (monetary condition=$5 loss; social condition=negative peer feedback).

The Social Doors Task is comprised of two sub-tasks: one involved social rewards and the other involved monetary rewards. Each was presented in separate runs with the order counterbalanced across participants (Fig. 2). This presentation method mitigates the influence of task switching costs while enabling a direct comparison of responses to both social and nonsocial rewards.

During the mock scan session (typically 1–3 weeks before the neuroimaging session), we took a picture of the participant and told them that other participants would complete a survey where they decided if they liked or disliked the current participant based on their photo. While completing the social task, participants viewed images of two peers and chose the one who they believed liked them based on their photo. Feedback following each trial reflected the peer's preference, with positive outcomes indicating liking and negative outcomes indicating disliking. During the monetary task, participants chose which of two doors contained a $10 prize. Feedback following each trial reflected the gain or loss of money, with positive outcomes indicating a win of $10 and negative outcomes indicating a loss of $5. Participants were told that the result from one monetary task trial would be randomly selected and added or subtracted from their bonus payments for participation. In both tasks, each session comprised 60 trials, evenly distributed between reward and loss outcomes. For all trials, participants had 3 s to make a decision and feedback was displayed for 1 second. Trials were separated by variable intertrial intervals ranging from 1.1 to 11.6 s, with a mean duration of 3.5 s.

#### 4.2.3 Trust task

**Fig. 3:**
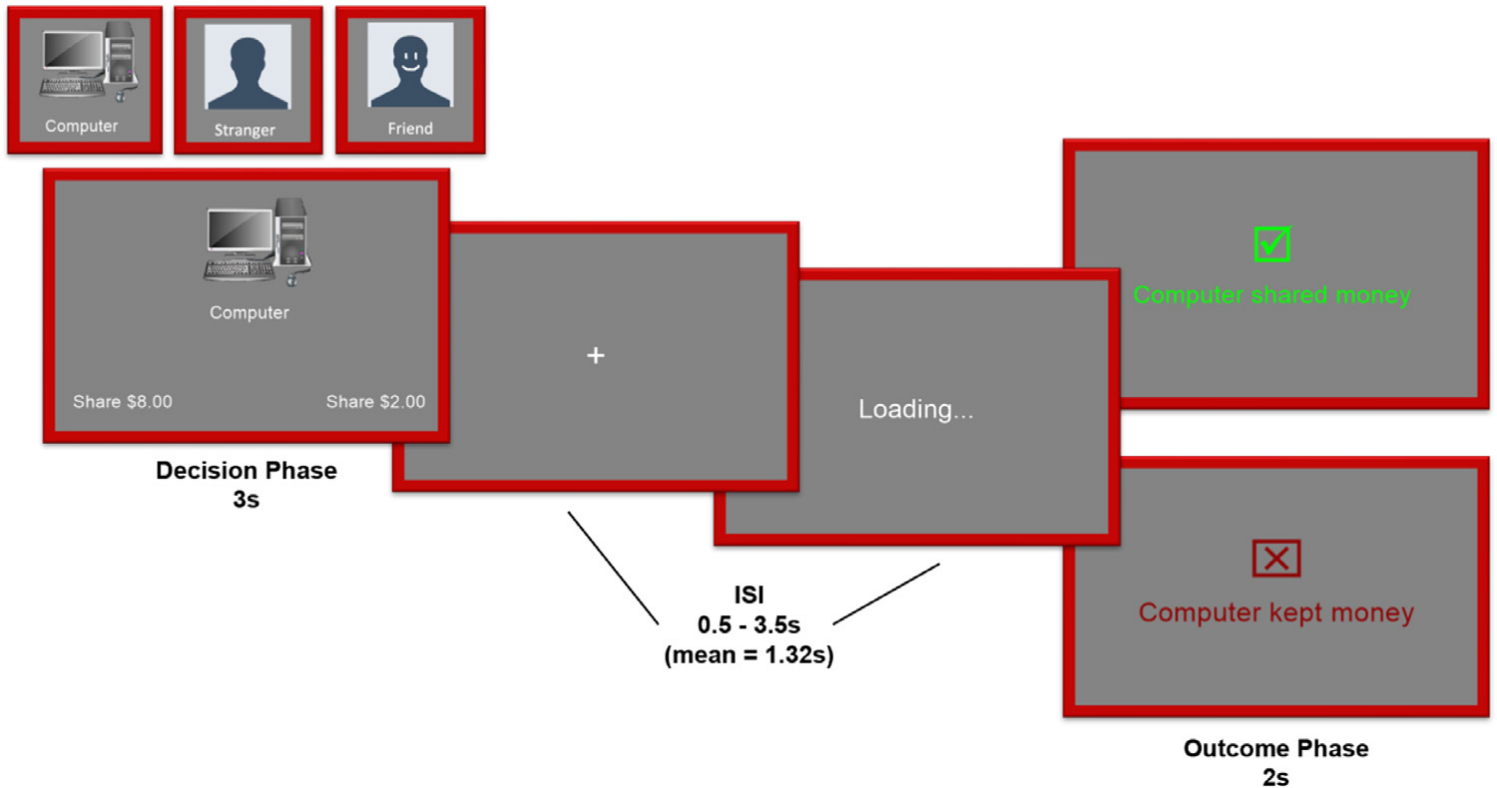
Trust Task. Participants play each round with one of three possible partners, a computer, a stranger, or their friend. During the decision phase, participants choose one of two possible amount to invest in their partner, which is tripled and given to them. The participant keeps the remainder. During the outcome phase, participants see whether their friend chose to share the tripled amount evenly with them or not.

In the trust task, participants invest a share of $8.00 (ranging from $0.00 to $8.00) in the partner they have been assigned for that trial (Fig. 3). That partner may be either their study partner, the computer, or a gender- and age-matched confederate. Their friend is the same person as in the Shared Reward task, and the confederate is a different person than in the Shared Reward task. The amount that the participant decides to invest in the partner is tripled, and the participant is told whether their partner shared the total amount evenly with them or kept it all for themselves.

#### 4.2.4 Ultimatum game task

**Fig. 4:**
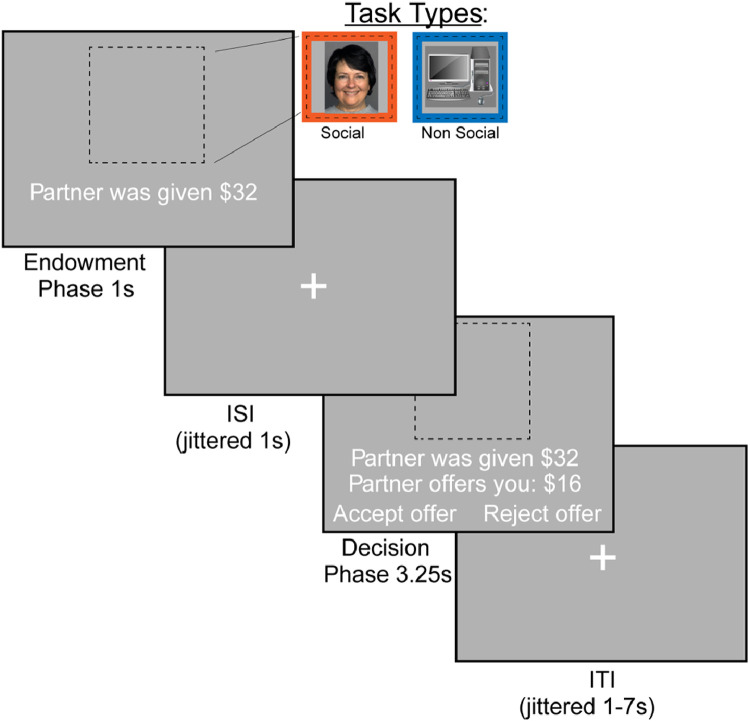
Ultimatum Game. Participants decided to accept or reject offers made by a social or nonsocial partner. If they rejected the offer, neither the participant nor their partner made any money. Participants were offered between 5 % and 50 % of the initial endowment.

In the ultimatum game, participants are made offers by two different partners (a stranger or a computer) and decide whether to accept or reject the offer (Fig. 4). When paired with a stranger, participants were informed that their partner made real choices in past games and that their choices may be used with future participants. Each stranger was experienced only once to maintain the one-shot design of the game. When paired with a computer, participants were informed that their choices were decided by an algorithm and the winnings would return to the general pool of lab funds. Participants are told that the partner received $16 or $32, and decided to offer to share 5 %, 10 %, 25 % or 50 % of this endowment with them. If they accept the offer, they both receive the money as the split proposes. However, if they reject the offer, neither the participant nor the partner will receive any money. Offers were calculated to be “fair” when they were 25 % or 50 % of the initial received amount and “unfair” when the participant was offered 5 % or 10 % of the initial endowment. Each trial consisted of the endowment phase (1 second), an interstimulus interval of 1–4 s (*M* = 2.4 s), decision phase of 3.25 s, and an intertrial interval of 1–7 s (*M* = 2.5 s).

#### 4.2.5 MRI data acquisition

We collected anatomical, diffusion, and functional images using a 3.0 Tesla Siemens PRISMA MRI scanner and a 20-channel head coil at the Temple University Brain Imaging and Research Center in Philadelphia, Pennsylvania, USA.

#### 4.2.6 Anatomical data

We collected two types of anatomical data to aid spatial normalization (T1w) and characterization of white matter hyperintensities (FLAIR). A T1w image was collected using a high-resolution magnetization-prepared rapid acquisition gradient echo (MPRAGE) sequence with in-plane acceleration (GRAPPA=2) with the following parameters: field of view (FOV) = 224 mm; matrix = 224×224; 1 mm^3^ isotropic resolution; 192 sagittal slices; repetition time (TR) = 2400 ms; echo time (TE) = 2.17 ms; flip angle = 8° These T1w images are essential for spatial normalization and anatomical location. They can also be used to provide estimates into brain age [17]. In addition, we also collected T2w fluid attenuated inversion recovery (FLAIR) images using in-plane acceleration (GRAPPA=3) and the following parameters: FOV = 220 mm; matrix = 256×256; 0.86 mm^3^ in-plane resolution; 47 axial slices (3 mm thick); voxel size = 0.86×0.86×3.00 mm; TR = 9000 ms; TE = 88 ms; flip angle = 150° These T2w FLAIR images are sensitive to white matter hyperintensities.

#### 4.2.7 Diffusion data

We collected diffusion data using a hybrid multi-shell diffusion weighted imaging sequence with in-plane acceleration (GRAPPA=2) and simultaneous multislice (multiband factor = 3) using parameters adapted from previous work [18]. Specifically, the diffusion-weighted imaging sequence involved 145 diffusion-weighted images. These images consisted of 6 *b* = 250 s/mm^2^, 21 *b* = 1000s/mm^2^, 24 *b* = 2000s/mm^2^, 30 *b* = 3250 s/mm^2^, 61 *b* = 5000 s/mm^2^ and 3 T2-weighted *b* = 0 s/mm^2^ acquisitions with 2 mm^3^ isotropic voxels (matrix = 120×120; 69 axial slices; TR = 2710 ms; TE = 84.6 ms; flip angle = 90°). We also collected spin-echo fieldmaps with anterior to posterior and posterior to anterior phase-encoding directions to quantify distortions. These spin-echo fieldmaps matched the geometry of the diffusion images and can be used to undistort the diffusion images during preprocessing.

#### 4.2.8 Functional data

We collected multi-echo fMRI data using a gradient echo-planar imaging (EPI) with four echoes (13.8, 31.54, 49.28, 67.02 ms) and simultaneous multislice (multiband factor = 3) with in-plane acceleration (GRAPPA = 2) and partial Fourier set to 7/8. This multi-echo sequence was collected with the following parameters: matrix = 80×80; 2.7 mm^3^ isotropic resolution; 10 % gap between slices; 51 axial slices; TR = 1615 ms. We collected both magnitude and phase components of the functional images, which may facilitate denoising and undistortion approaches [4]. We note that our initial scans used a lower flip angle (20°) with the idea that this lower flip angle would reduce physiological noise [6]; however, we increased the flip angle (50°) following discussion generated by a presentation of preliminary data at the Organization for Human Brain Mapping, concluding that the logic for improved tSNR may not hold for longer echoes (see Limitations). In addition, we also collected single-band reference images with each functional run of multiband data to improve motion correction and registration. Finally, we also collected gradient echo B0 fieldmaps to unwarp and undistort functional images (TR: 645 ms; TE1: 4.92 ms; TE2: 7.38 ms; matrix 80×80; voxel size: 2.7 × 2.7 × 2.7 mm; 54 slices, with 10 % gap; flip angle: 60°).

## Limitations

**Fig. 5:**
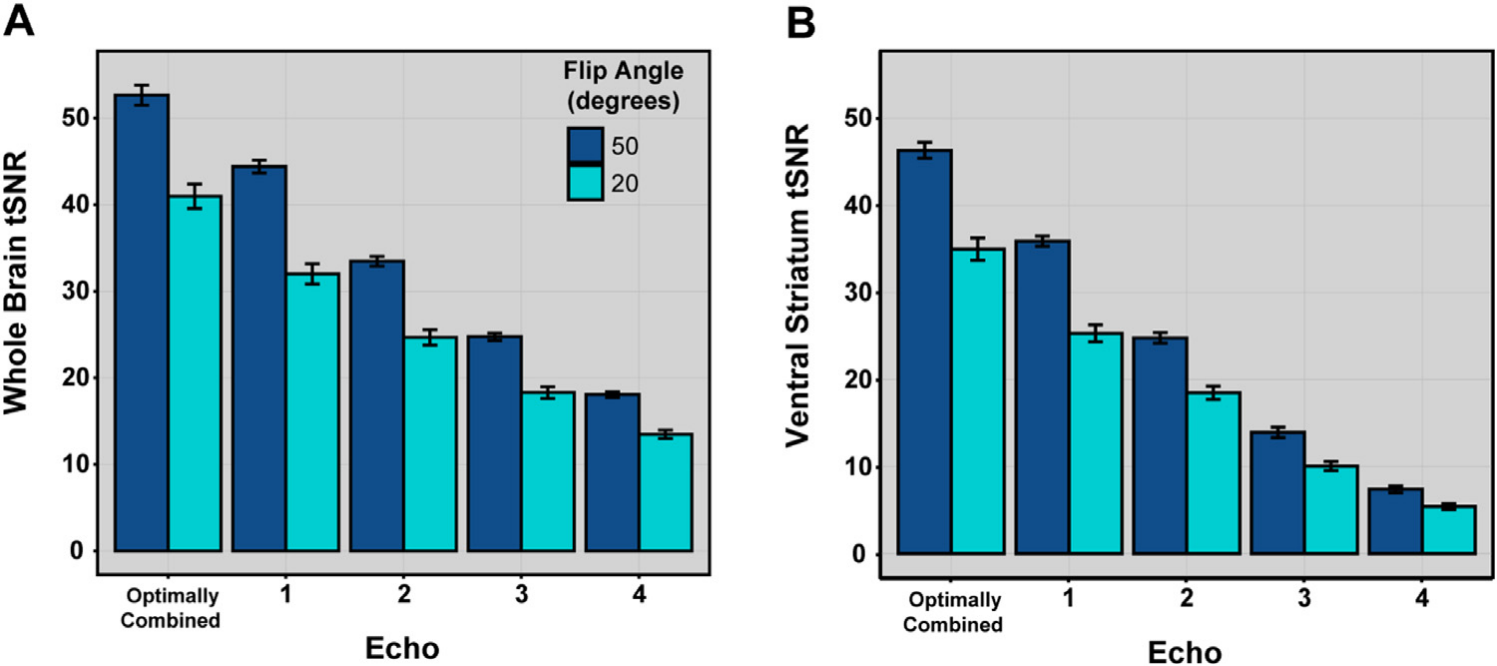
Panel A and B display the temporal signal-to-noise ratio (tSNR) for each echo (1 to 4) and flip angle (50° and 20°) that were collected during scanning procedures for whole brain and ventral striatum, respectively. tSNR values for echoes 1 to 4 were extracted from native space preproc_bold.nii.gz images. The optimally combined values were extracted from standard space (MNI152Lin6Asym) preproc_bold.nii.gz images. Error bars represent standard error of the mean.

This fMRI dataset has some limitations that may impact its reuse. First, it is important to note that age-related differences in fMRI responses may be confounded with artifacts or data quality, thus we encourage users to consider appropriate covariates in their analyses [19]. We also note that this dataset was collected using two flip angles. For our initial 47 participants, we used a 20-degree flip angle that was intended to reduce physiological noise [6]. However, after presenting preliminary results at the 2023 meeting of the Organization for Human Brain Mapping, it became clear to us that a higher flip angle (i.e., 50°) may help improve signal in our longer echoes, which may help denoising efforts [20] and maximize the utility of multi-echo data [6]. To quantify this effect within the current dataset, we examined temporal signal noise to ratio (tSNR) across the whole brain and also the ventral striatum. We found that the larger flip angle is associated with increased tSNR for the optimally combined data and each of the echoes (all *P*s < 0.0002, uncorrected; Fig. 5). Thus, users of this dataset may wish to include covariates that account for the change in flip angle and tSNR.

In addition to individual differences in data quality, we also note that the project is ongoing, and we expect to release additional data at the conclusion of the project. Nevertheless, we believe this preliminary release of over 100 scans of multi-echo fMRI data is important because of its size and unique focus on social and nonsocial reward processing, receipt of reward in social contexts, and social decision making.

## Ethics Statement

All participants provided written informed consent to engage in the study and share their de-identified data publicly. The study was approved by the Institutional Review Board at Temple University (Philadelphia, Pennsylvania, USA) under Protocol Number 28,455, and it was conducted in accordance with the Declaration of Helsinki.

## CRediT Author Statement

**David V. Smith**: Conceptualization, Methodology, Software, Formal Analysis, Data Curation, Writing - Original Draft, Writing - Review & Editing, Supervision, Project Administration, Funding acquisition. **Cooper J. Sharp**: Software, Formal Analysis, Investigation, Data Curation, Writing - Original Draft, Writing - Review & Editing, Visualization. **Abraham Dachs**: Investigation, Writing - Review & Editing. **James B. Wyngaarden**: Software, Investigation, Writing - Original Draft, Writing - Review & Editing, Visualization. **Daniel Sazhin:** Methodology, Software, Investigation, Writing - Original Draft, Writing - Review & Editing, Visualization. **Jen Yang:** Investigation, Writing - Original Draft, Writing - Review & Editing, Visualization. **Melanie Kos:** Investigation, Writing - Original Draft, Writing - Review & Editing, Visualization. **Tia Tropea:** Investigation, Writing - Review & Editing. **Ishika Kohli:** Investigation, Writing - Review & Editing. **John A. Clithero:** Writing - Review & Editing. **Ingrid Olson:** Methodology, Writing - Review & Editing. **Tania Giovannetti:** Conceptualization, Writing - Review & Editing. **Dominic S. Fareri**: Conceptualization, Methodology, Software, Writing - Review & Editing. **Johanna M. Jarcho**: Conceptualization, Methodology, Writing - Original Draft, Writing - Review & Editing.

## Data Availability

Social relationships change across the lifespan as social networks narrow and motivational priorities shift. These changes may affect, or reflect, differences in how older adults make decisions relate (Original data) (OpenNeuro).
